# Isolated arthroscopic treatment of intra-articular pathologies in mild hip dysplasia: a short-term case control study

**DOI:** 10.1186/s40634-021-00428-w

**Published:** 2021-12-03

**Authors:** Enrico Tassinari, Federica Mariotti, Francesco Castagnini, Stefano Lucchini, Francesco Perdisa, Giovanni Bracci, Monica Cosentino, Barbara Bordini, Francesco Traina

**Affiliations:** 1 Ortopedia-Traumatologia e Chirurgia Protesica e dei reimpianti di Anca e di Ginocchio, IrCCs Istituto Ortopedico Rizzoli, Via Pupilli 1, 40136 Bologna, Italy; 2 Laboratorio di Tecnologia Medica, IrCCs Istituto Ortopedico Rizzoli, Bologna, Italy

**Keywords:** Hip arthroscopy, Borderline dysplasia, Femoro-acetabular impingement, Capsular plication

## Abstract

**Purpose:**

The aim is to compare the results of isolated hip arthroscopy in patients with borderline dysplasia with Lateral center edge angle (LCEA) between 18° and 25° with a control group of patients with normal LCEA (> 25°).

**Methods:**

Fifty hip arthroscopies performed in 45 patients were retrospectively evaluated. Exclusion criteria were: age > 40, hip arthritis > grade 2 according to Tonnis classification, femoral head avascular necrosis, pediatric’s orthopaedics conditions and true dysplasia with LCEA < 18°.Two groups were identified: group A with 15 hips with LCEA between 25° and 18° and Group control B made of 35 hips with LCEA > 25°.

**Results:**

The groups were homogeneous for demography and pre-operative WOMAC and HOOS. Osteoplasty for CAM were performed in 100% of patients in both groups, only in 12 hips (34.4%) in group B we had both femoral and acetabular osteoplasty. Labral repair was performed in 86% of patients in group A, in 60% of patients in group B, capsular plication in 93% of group A, in 5% of case of group B. WOMAC and HOOS statically significant improved in both groups at final follow-up (24 months). No cases in both groups required conversion to total hip arthroplasty.

Clinical outcomes of study group were comparable to the control group.

**Conclusion:**

Even if the present small series is not conclusive, we suggest isolated arthroscopic management of patients with FAI and LCEA between 18° and 25°, but capsular plication and careful labral management are strongly recommended.

**Level of evidence:**

Level IV.

## Introduction

Hip arthroscopy is considered the technique of choice in the treatment of femoro-acetabular impingement (FAI) or labral tears, but its use in the treatment of mild hip developmental dysplasia (DDH) is under debate [8]. The pathomechanics of hip dysplasia implies acetabular undercoverage with excessive load on the acetabular rim and premature development of hip osteoarthritis [20]. On the contrary, FAI is characterized by acetabular overcoverage, nonetheless both these morphologic alterations can occur in the same patient [1].

Pelvic reorientation osteotomies have been traditionally adopted for the treatment of symptomatic moderately dysplastic hips, with the goal to modify the improper load conditions, thus preventing premature degenerative changes. Pelvic osteotomies are technically demanding, complications may be expected in as many as 15% of cases and noticeable rates of conversion to total hip arthroplasty (THA) have been reported in some series [1, 9, 12]. Furthermore they are invasive procedures and not always well accepted especially by younger patients. On the other side, the adoption of arthroscopic approaches in mild DDH is controversial with unpredictable outcomes [8]. In patients with lateral center edge angle (LCEA) between 18° and 25° the labrum and capsule seem to have a relevant role in maintaining hip stability, and hip arthroscopy should play a relevant role in preventing chondrolabral degeneration in this subset of patients [2]. However, meticulous surgical techniques is mandatory in such cases, because iatrogenic instability, joint subluxation and premature chondrolabral derangement have been reported [1, 4, 6, 16].

The aim of the present study is to compare the clinical results of hip arthroscopic in patients with borderline dysplasia (LCEA between 18° and 25°) with a control group of patients with normal LCEA (> 25°). Our hypothesis is that hip arthroscopy can be a valuable alternative in the treatment of symptomatic patients with borderline dysplasia.

## Materials and methods

We retrospectively evaluated 50 hip arthroscopies (45 patients, 5 bilateral hips) performed consecutively in a single center between 2015 and 2018 for Femoro-Acetabular Impingement. All surgeries were performed by a single surgeon that performed in his career a number of operations that is far larger than the number suggested to reach the learning curve plateau. Patients were clinically and radiographically followed after the hip arthroscopy, in a prospective fashion. Informed consent was obtained by each patient in the study.

Inclusion criteria were:

- diagnosis of symptomatic femoroacetabular impingement not responding to conservative treatment
- diagnosis of symtomatic labral tears
- mild hip dysplasia with LCEA beetween 18°- 25°

Exclusion criteria were:

- age > 40 years,
- hip arthritis > grade 2 according to Tonnis classification,
- femoral head avascular necrosis and pediatric’s orthopaedics conditions such as Phertes disease and slipped capital femoral epiphysis,
- true dysplasia with LCEA < 18°
- breaking in Shenton line
- lateralization of the femur > 1 cm
- excessive coxa valga (neck-shaft angle < 115°)

Radiological investigations were performed in all the patients before surgery using anteroposterior radiographs of the pelvis and lateral Dunn 45° view. Preoperative standard MRI was obtained in order to study cartilage status, labral injuries and soft tissues conditions.

The post-surgical radiographic assessment included an anteroposterior radiograph of the pelvis every 6 months in order to evaluate arthritis progression and standard MRI only once, at 6 months in order to evaluate soft tissue conditions and cartilage status.

The following measurements were performed as described by previous papers^10^:

- LCEA
- Tonnis angle
- Type of FAI (Cam, Pincer or combined)
- grade of hip arthritis (according to Tonnis classification),
- joint space narrowing, considering as cut off 2 mm of residual joint space,
- crossover sign and the prominence of the ischial spine.

The patients were clinically assessed in the pre-surgical and post-surgical settings using HOOS and WOMAC score.

Pre-surgical demographic and radiographic data are specified in the Table 1.

**Table 1:** Demographic and radiological pre-surgical data of both groups

	STUDY GROUP A	CONTROL GROUP B
Number of cases	15 patients	35 (30 patients)
Mean age	31 (16–39)	29.1 (19–39.5)
Female: Male (n)	5: 10	9: 21
Side (Right: Left) (n)	7: 8	23: 13
Mean Body Mass Index (Kg/m^2^)	23.94 (21.3–28.7)	23.6 (18.6–31)
Cam impingement (n)	12	10
Pincer impingement (n)	0	4
Combined impingement (n)	3	21
Mean Lateral Center-Edge Angle(°)	23,35° ± 2,34 (18°-25°)	34,22° ± 4,58 (27,5°-44°)
Mean Acetabular Inclination (°) (Tonnis Angle)	8.3° (3.0°-13.5°)	6.0° (2.5°-11.6°)
Tonnis Arthritis Grade (0, 1, 2)	2, 11, 2	10, 24, 1
Joint space narrowing (< 2 mm)	0	0
Crossover Sign (n)	3	18
Prominence of the Ischial Spine (n)	3	19
Mean acetabular chondropaty (Outerbridge scale)	2,2	1,08
Mean femoral chondropaty (Outerbridge scale)	1,5	0,97

The hips were divided in two major groups: in the study group or Group A, we considered patients with an LCEA between 25° and 18° (borderline dysplasia range as identified by Bird et al. [5]), and in the control group or Group B, there were patients with a LCEA > 25° (Table 1).

### Arthroscopic procedures

In all 15 hips (100%) in group A and in 23 hips (65.7%) in group B we performed isolated femoral head osteoplasty, while in 12 hips (34.4%) in group B received both femoral and acetabular osteoplasty. No acetabular osteoplasty was performed in group A.

Acetabular microfractures were performed when Outerbridge grade 4 ostheocondral lesions were found, this procedure. This was performed in 6 cases (40%) in group A and in 3 cases (8.6%) in group B. Capsular suture was performed in 14 hips (93.3%) in group A and in 2 hips (5.7%) in group B. This was performed at the end of the others procedure and without traction. Concerning labral tears treatment, 2 hips (13.3%) underwent labral shaving/debridement and 13 (86.7%) had labral repair with suture anchors in group A. In group B 7 hips (20.0%) underwent labral shaving/debridement and 21 hips (60.0%) had labral repair (see Table 2).

**Table 2:** Arthroscopic procedures performed in both groups

	STUDY GROUP A	CONTROL GROUP B
Number of Patients	15	30 (5 bilateral)
Femoral Osteoplasty	15 (100%)	23 (65.7%)
Femoral + Acetabular Osteoplasty	0	12 (34.3%)
Acetabular Microfractures	6 (40%)	3 (8.6%)
Capsular Plication	14 (93.3%)	2 (5.7%)
Labral shaving	2 (13.3%)	7 (20%)
Labral suture	13 (86.7%)	21 (60%)
Additional Procedures	0	2 Sinoviectomy (5.7%) 2 Ileopsoas Release (5.7%)

### Statistical analysis

Patient demographics and the other interventions characteristics were analyzed using descriptive statistics, such as means, medians, ranges, and percentages. Values were compared using non parametric tests as Mann-Whitney test (M-W test), Chi square test, Fisher test. Differences between pre and post values were compared using Wilcoxon nonparametric test for two paired groups. The threshold for significance was *p* = 0.05.

All statistical analyses were performed using SPSS 14.0, version 14.0.1 (SPSS Inc., Chicago, IL) and JMP, version 12.0.1 (SAS Institute Inc., Cary, NC, 1989–2007).

## Results

Both groups of patients resulted homogeneous for demographic parameters and preoperative WOMAC and HOOS. Radiographic parameters also were similar in both groups infact Tonnis arthritis grading in group A was found as Grade 0 in 2 hips (13.3%), Grade 1 in 11 hips (73,3%) and Grade 2 in 2 hips (13.3%). In group B 10 hips (28.6%) were Grade 0, 24 hips (68.6%) Grade 1 and 1 hip (2.9%) Grade 2. There were no hips with joint narrowing < 2 mm in both groups.

Instead intra-articular conditions such as type of FAI and cartilage damage where statically significantly different among the two groups: Femoroacetabular Impingement was Cam type in 12 hips of group A and combined CAM and PINCER in 3 hips in the same group, while in B group it was Cam type in 10 hips, combined FAI in 21 hips and PINCER type in 4 hips.

Acetabular Chondropathy according to arthroscopic Outerbridge scaling in group A was found ≤2 in 8 hips (53,3%) and ≥ 2 in 7 hips (46,7%), while in group B was found ≤2 in 32 hips (91.4%) and ≥ 2 in 3 hips (8.6%).

Femoral Chondropathy in group A was found ≤2 in 14 hips (93.3%) and ≥ 2 in 1 hip (6.7%), while in group B was found ≤2 in 34 hips (97.1%) and ≥ 2 in 1 hip (2.9%).

### Clinical outcomes

Preoperative mean HOOS was 72.4 ± 20.6 (41–107) in group A and 65.3 ± 14.2 (45–112) in group B (M-W test 0.18). Preoperative mean WOMAC was 50% ± 10 (40%–70%) in group A and 50% ± 10 (40%–60%) in group B (M-W test 0.96).

Postoperative mean HOOS at final follow up (24 months) resulted 24.2 ± 16.4 (12–80) in group A and 28.2 ± 8.5 (16–45) in group B (0.009 M-W test). Postoperative mean WOMAC at 24 months of follow up resulted 70% ± 10 (40%–80%) in group A and 80% ± 0 (60%–80%) in group B (Table 3).

**Table 3:** Clinical outcomes pre and postoperative in both groups

	STUDY GROUP A	CONTROL GROUP B	*P* value
HOOS pre-op			0.18
*Mean [sd]*	72.4 [20.6]	65.3 [14.2]	*(M-W test)*
*(min-max)*	(41–107)	(45–112)	
WOMAC pre-op			0.96
*Mean [sd]*	50% [10%]	50% [10%]	*(M-W test)*
*(min-max)*	(40%–70%)	(40%–60%)	
HOOS post-op			0.009
*Mean [sd]*	24.2 [16.4]	28.2 [8.5]	*(M-W test)*
*(min-max)*	(12–80)	(16–45)	
WOMAC post-op			0.82
*Mean [sd]*	70% [10%]	80% [0%]	*(M-W test)*
*(min-max)*	(40%–80%)	(60%–80%)	

In both study and control groups there was a statically significant improvement of postoperative HOOS and WOMAC score in comparison to the pre-operative values (*p* < 0.001 Wilcoxon test). The improvement in the two groups were comparable at 24 months from surgery.

There was no revision arthroscopy nor conversion to total hip arthroplasty (THA) in both groups at the final follow up.

## Discussion

Hip arthroscopy has been widely accepted as a less invasive surgical technique to treat a variety of pre-arthritic conditions, ranging from FAI to labral tears, chondral lesions and loose bodies, but the role of arthroscopic surgery in the management of mild dysplastic hips is under debate [3, 13]. This is a particular subset of patients that may be both suitable for arthroscopy and periacetabular osteotomies [6].

The treatment of patients with an LCEA between 18° and 25° is nowadays controversial since there is no agreement on the critical value of LCEA where bony correction is mandatory, and arthroscopic surgery has been reported to significantly improve symptoms in borderline dysplastic hips if associated with labral repair and careful capsular closure [7, 14]. As a matter of fact, less evidence-based data are available about the outcomes of such patients, and early reports considered borderline dysplasia to be a relative contraindication for hip arthroscopy [13]. Besides, the concept of LCEA to define hips labeled as “mild” or “borderline” dysplastic seems to be simplistic, since other radiological parameters should be taken into consideration [14].

**Fig. 1:**
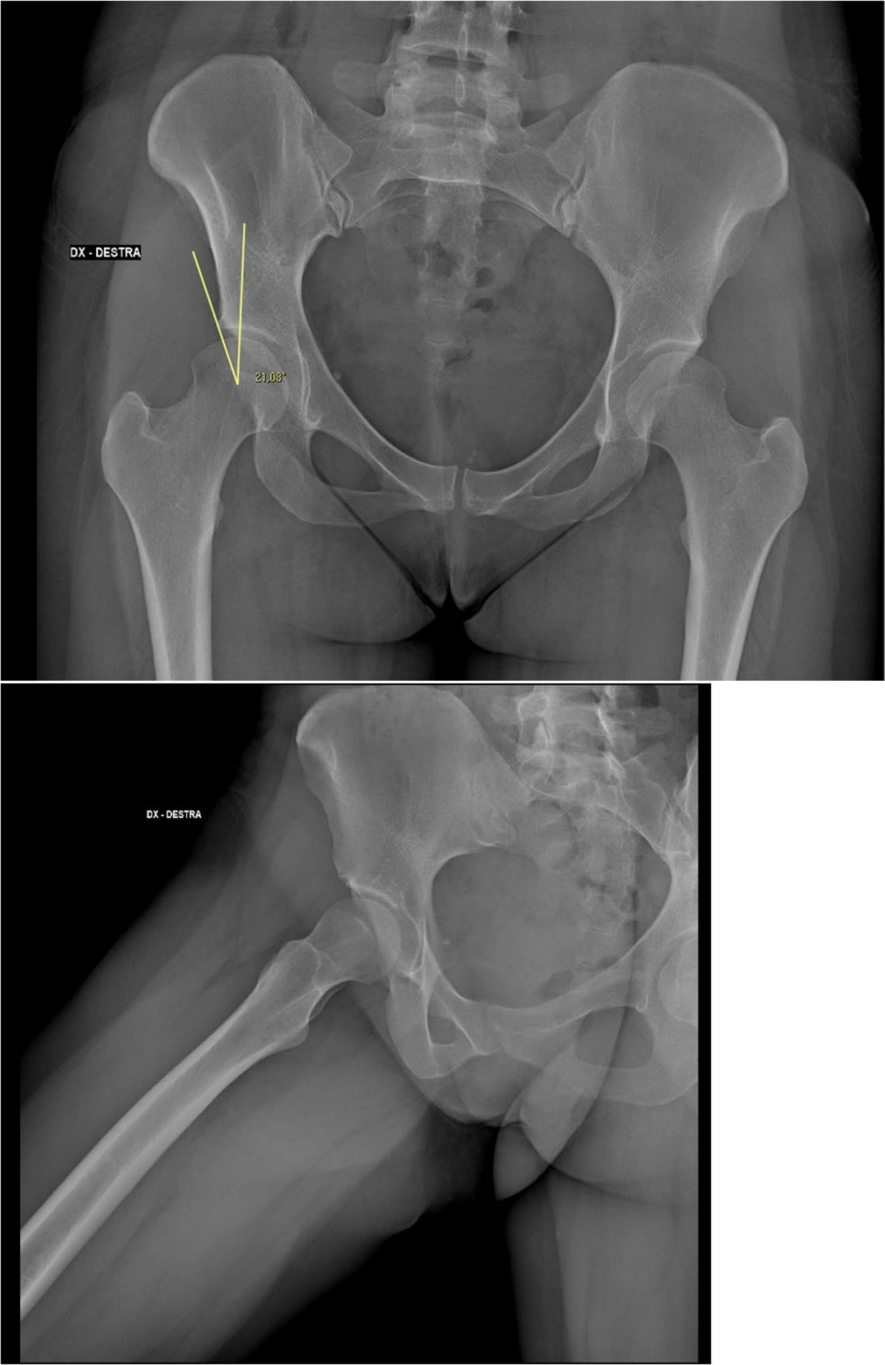
Pre-operative X-Ray evaluation of a mild displastic patient: Anteroposterior of the pelvis and lateral Dunn 45° view radiographs of a 22 years old female included in group A (LCEA of 21.08°) with a CAM type impingement of the right hip

**Fig. 2:**
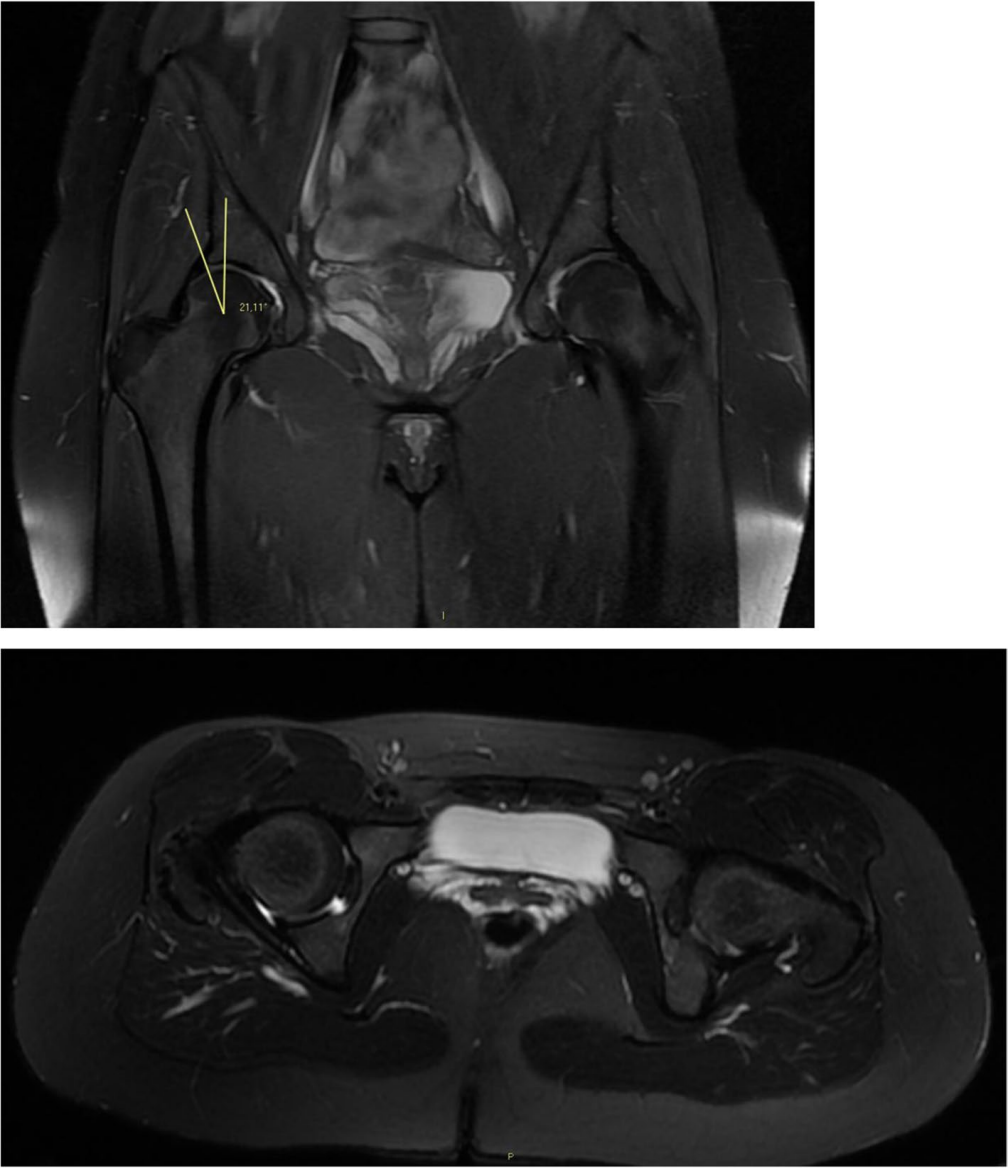
MRI evaluation of a group A patient: Coronal and Axial MRI view of the previous 22 years old female showing no chondral damage and mild dysplasia of the right hip

The aim of the present study was to evaluate the outcomes of hip arthroscopy in patients with borderline dysplasia (LCEA between 18° and 25°, Figs. 1 and 2). The results of our series demonstrated that hip arthroscopy can be a valuable alternative in this subset of patients, if associated with capsular suture and careful labral management.

Although a significantly larger number of osteochondral lesions have been encountered and treated in the study group (see Table 2), clinical outcomes are comparable to the control group of normal hips (LCEA greater than 25°). As it could be expected, a significantly lower rate of pincer FAI has been recorded in the study group. Besides, capsular plication was significantly more frequent in the study group (see Table 2). There was no conversion to THA in both groups.

Besides, concerns have arisen about the adequacy of LCEA in defining borderline dysplasia, because acetabular undercoverage should be evaluated in the anterior, posterior and lateral regions by using additional radiographic parameters, such as acetabular inclination angle of Tönnis, the anterior center-edge angle, the anterior wall index (AWI) and posterior wall index (PWI), and the femoral epiphyseal acetabular roof (FEAR) index [10, 11, 14, 15, 17–19]. As it was stated above, a thorough radiographic assessment of acetabular coverage should be implemented in the setting of hip dysplasia, because failure of arthroscopic approaches in borderline dysplastic hips may be due to an inadequate evaluation of proximal femoral anatomy. In this scenario, hips classified as “borderline” or “mild” dysplastic on the basis of the LCEA should be probably scheduled for hip arthroscopy only if other radiographic parameters (particularly the FEAR index) fall into the normal range value. In the case of multiple abnormal radiographic values, periacetabular osteotomy (PAO) can be considered.

The results of our series are in line with those of the recent literature about this topic. Domb et al. [6] demonstrated that patients with borderline dysplasia can achieve similar clinical improvements after undergoing hip arthroscopy with subsequent capsular repair. Similarly, Beck et al. [3] showed that, at least in the short term, patients with borderline dysplasia undergoing hip arthroscopy with capsular plication and careful labral management can anticipate same clinical outcomes when compared with their counterparts with normal LCEA. Finally, in a large multicenter study, Matsuda and coworkers demonstrated that LCEA did not influence outcomes of primary hip arthroscopy performed in borderline dysplastic patients [13]. Our results confirm those findings, and support the role of arthroscopy in borderline dysplasia, if capsular closure is routinely performed to avoid iatrogenic instability. This study has some relevant limitations. First of all, it is retrospective in nature and used a short-term follow-up data. Secondly, the small number of patients especially in the study group. Thirdly there is not enough statistical power.

## Conclusions

**Fig. 3:**
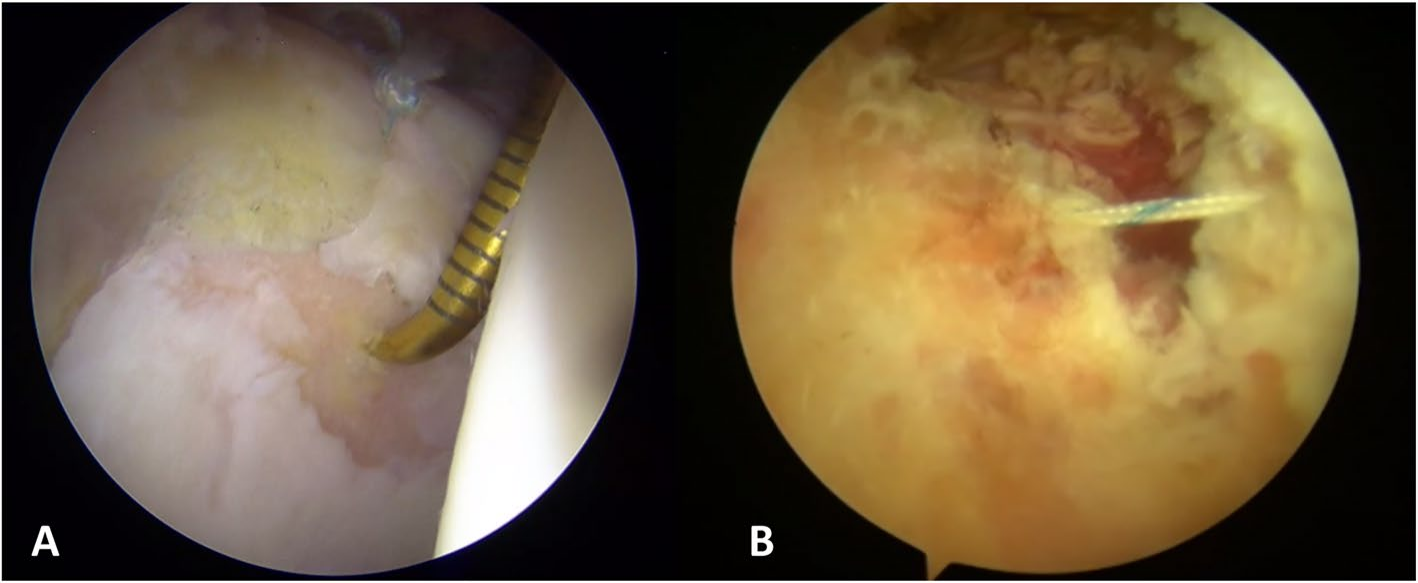
Arthroscopic view from antero-lateral portal of a borderline displastic hip: acetabular Chondropathy area grade 4 according Outerbridge are detected and treated with microfractures after performing a labral suture with one anchor (**A**). Capsular plication (**B**) is performed at the end of the intrarticular procedures

On the basis of our results, clinical outcomes of study group were comparable to the control group of normal hips, we suggest arthroscopic management of patients with FAI and LCEA between 18° and 25°, but capsular plication and careful labral management are recommended (Fig. 3). The thorough radiographic evaluation of acetabular coverage is crucial to identify those patients more suitable for PAO.

## Abbreviations

FAI: Femoro-acetabular impingement
DDH: Developmental dysplasia of the hip
LCEA: Lateral center edge angle
THA: Total hip arthroplasty
HOOS: Hip disability and Osteoarthritis Outcome Score
WOMAC: Western Ontario and MCMaster University Arthtritis Index
MRI: Magnetic resonance imaging
PAO: Periacetabular osteotomy
AWI: Anterior wall index
PWI: Posterior wall index
FEAR: Femoral epiphyseal acetabular roof index
